# Evaluating and Improving upon Ecuador’s Adolescent Pregnancy Prevention Policies in an Era of Increased Urgency

**DOI:** 10.5334/aogh.3030

**Published:** 2020-09-01

**Authors:** Keren Herrán, Iván Palacios

**Affiliations:** 1 University of Maryland, Baltimore County, US; 2 Universidad San Francisco de Quito, EC

Latin America and the Caribbean (LAC) is the only region worldwide in which incidence rates of pregnancy for adolescents younger than 15 years of age is increasing [1]. With 111 births per 1,000 adolescent girls (15–19 years old), Ecuador has the highest adolescent fertility rate among all LAC countries [2]. These figures are alarming given the significant medical, social, and economic inequalities exacerbated by adolescent pregnancy (AP) [3].

Given the prior association between disease outbreaks and AP, the COVID-19 pandemic presents an urgent international concern for adolescent reproductive health. For instance, the Ebola outbreak in Sierra Leone indirectly caused a 65% increase in AP within target communities [4]. School closures and economic downfall following periods of quarantine augmented sexual exploitation. Therefore, COVID-19 underlines the need to evaluate and learn from prior health policy in strategizing more effective AP prevention legislation. Reviewing Ecuador’s attempts to reduce AP, first through the implementation of differentiated services for adolescents, then via establishing an integrated lifecycle model, and lastly, their current strategy involving gendered violence, demonstrates how each plan has varied in effectiveness yet collectively failed to create an upstream approach that builds human capital.

First, examining Ecuador’s Ministry of Health’s establishment of differentiated services for adolescents (SADAs) in 2007 reveals that solely concentrating on providing more comprehensive sexual and reproductive health instruction can be an unnecessary and reductionist approach. SADAs was formed based on the assumption that adolescent pregnancy was the result of health education knowledge gaps that could be bridged by increasing access to trustworthy, non-condescending, approachable health professionals who only served adolescents. The program provided 1,061 health professionals with sensitization workshops and specialized medical training for working with adolescents [5]. However, the demographic census released before SADAs in 2005 reported that 96% of women in Ecuador between the ages of 15 and 49 expressed they already knew of information regarding contraceptive methods and that 95.5% of them were aware of modern preventative options available to them [6]. Therefore, by focusing on the individual, SADAs failed to recognize and address the larger systemic driving forces of AP.

Second, Ecuador’s reversal of approach to centralizing adolescent health services in 2011 exemplifies how an abrupt shift in how health information is dispensed does not ensure greater efficacy of AP prevention. Policymakers concluded that the fragmented care structure created by SADAs, with its emphasis on providing specialty services to adolescents, actually worsened inequalities in health services. Thus, the new Model of Comprehensive Family, Community, and Intercultural Health Care (MAIS-FCI) emphasized the equal level of care throughout each stage of an individual’s life cycle while also emphasizing that the adolescent population, in particular, continues to be intensely equipped with medical information [7]. However, the lack of guidance at the local level during this policy transition led to a regression in community health clinics’ quality of services. Due to added workload requirements and confusion surrounding which competencies they were to continue under the new framework, health personnel had difficulty sustaining adolescent-targeted care. Most concerning, Ecuador had continued using the promotion of health information as the main AP prevention strategy.

Third, Ecuador’s latest adolescent pregnancy prevention tactic, the Intersectoral Policy for the Prevention of Pregnancy in Girls and Adolescents, at last, offers a plan that can serve as a starting point in the nation’s development of solutions that do not simply fault adolescents for AP, but rather aim to mitigate the social/cultural dynamics that act as an impetus. For example, one of the scheme’s main goals is to improve the justice system such that it is easier for adolescents to file charges against sexual predators [8]. Hopefully, future evaluations of this rights-based approach will find it to be successful in decreasing AP especially among girls under 15 years, whose rates of AP in LAC have been increasing.

To diminish AP in the era of COVID-19, Ecuador should design interventions focused on tackling social/cultural barriers and economic inequities. Only 11% of adolescent girls from wealthy Ecuadorian households give birth, whereas, for girls in impoverished households, this rate reaches 28% [9]. Adolescents living in communities characterized by social isolation and limited job opportunities may view early parenthood as a means of ascending in social status. If a greater effort is placed into mandating intense career-counseling and creating job opportunities, disadvantaged adolescent boys and girls may overcome their neighborhood’s culture of despair. Given viable aspirations, they will be more likely to obtain economic mobility and delay parenthood [10]. Empowering girls economically may also reduce their dependency on male partners and strengthen their ability to refuse partaking in unprotected sex.
